# Criminal Justice Reform as HIV and TB Prevention in African Prisons

**DOI:** 10.1371/journal.pmed.1001215

**Published:** 2012-05-08

**Authors:** Katherine W. Todrys, Joseph J. Amon

**Affiliations:** Human Rights Watch, New York, New York, United States of America

## Abstract

Katherine Todrys and Joseph Amon argue for criminal justice system reforms in sub-Saharan Africa to reduce HIV and TB transmission in prisons and to guarantee detainees' human rights and health.

**Summary Points**

- HIV and tuberculosis (TB) prevalence in sub-Saharan Africa is higher in prison than in non-prison populations due, in part, to on-going transmission related to overcrowding and a lack of adequate prevention and treatment services.
- Almost all prisoners eventually leave prison and, along with visitors and prison officers, represent a potential bridge for disease transmission between prison and community populations.
- Thousands of individuals are detained in African prisons unjustly and unnecessarily, including spending long periods in pretrial detention, because of weak criminal justice systems.
- Alleviating overcrowding by increasing the availability of non-custodial alternatives including community service and bail, and improving access to legal representation, are essential public health measures for HIV and TB prevention and control in African prisons.
- African governments and international health donors should fund justice initiatives and other structural interventions to address HIV and TB in prisons and the general population in Africa.

## Background

HIV prevalence in sub-Saharan African prisons has been estimated at two to 50 times that of non-prison populations [1], while average tuberculosis (TB) incidence in prisons worldwide has been estimated at more than 20 times higher than in the general population [2]. Overcrowding—resulting in and exacerbating food shortages, poor sanitation, and inadequate health care—contributes to the spread and development of disease [3],[4]. Minimal ventilation, poor isolation practices, and a significant immune-compromised population also facilitate the transmission of TB and the development of TB disease [5].

Prison health care in Africa is under-resourced, and increased funding is needed to ensure adequate treatment is available, including antitretroviral (ART) therapy as treatment for HIV, and for HIV and TB prevention [6],[7]. However, even when ART is available, certain classes of prisoners such as foreign nationals may not be receiving treatment [8]. In addition, structural barriers, such as laws criminalizing “sodomy,” policies or practices limiting bail, and justice system problems resulting in long delays in accessing courts, impede prevention efforts and complicate the provision of care.

Prisons throughout sub-Saharan Africa are often filled far beyond their capacity. The prison populations in Burundi, Cote d'Ivoire, Kenya, Mali, Uganda, and Zambia are over 200% of capacity; in Benin, it is over three times design capacity (Table 1). Overcrowding can be so severe that inmates may be forced to sleep seated, standing, or in shifts, in cells with little ventilation. These conditions violate international standards [9] and may rise to the level of cruel, inhuman, or degrading treatment [10].

**Table 1:** Approximate prison population, capacity, and percentage of remanded prisoners in Sub-Saharan African prisons [39].

Country	Prison Population (Year)	Official Capacity (Year)	Remand Prisoners, Percent of Total Prison Population (Year)
**Angola**	16,183 (2009)	6,000 (2002)	59% (2003)
**Benin**	6,908 (2010)	1,900 (2006)	75% (2010)
**Botswana**	5,216 (2009)	3,967 (2009)	17% (2009)
**Burkina Faso**	5,238 (2010)	2,660 (2009)	48% (2010)
**Burundi**	9,844 (2010)	4,050 (2010)	63% (2010)
**Cameroon**	23,368 (2009)	15,250 (2009)	61% (2008)
**Cape Verde**	1,300 (2010)	N/A	37% (1999)
**Central African Republic**	1,320 (2010)	6,000 (2002)	N/A
**Chad**	3,416 (2005)	N/A	58% (2005)
**Comoros**	130 (2010)	N/A	50% (1998)
**Congo**	1,000 (2010)	N/A	70% (2008)
**Cote d'Ivoire**	11,143^a^ (2008)	4,871^a^ (2007)	29% (2007)
**Democratic Republic of the Congo**	30,000 (2004)	N/A	N/A
**Equatorial Guinea**	N/A	N/A	N/A
**Eritrea**	N/A	N/A	N/A
**Ethiopia**	112,361 (2009–2010)	N/A	14% (2009–2010)
**Gabon**	2,750 (2006)	N/A	40% (2006)
**Gambia**	780 (2009)	780 (1999)	30% (2009)
**Ghana**	13,573 (2010)	7,875 (2010)	29% (2009)
**Guinea**	N/A	N/A	N/A
**Guinea-Bissau**	N/A	90 (2010)	N/A
**Kenya**	52,000 (2012)	22,000 (2012)	43% (2009)
**Lesotho**	2,498 (2010)	2,910 (2010)	18% (2010)
**Liberia**	1,524 (2010)	750 (2007)	85% (2010)
**Madagascar**	18,647 (2010)	10,199 (2009)	43% (2010)
**Malawi**	11,672 (2010)	6,070 (2009)	19% (2009)
**Mali**	5,041 (2011)	3,000 (2009)	89% (2004)
**Mauritania**	1,700 (2010)	800 (2005)	41% (2010)
**Mauritius**	2,354 (2009)	2,194 (2008)	30% (2008)
**Mozambique**	16,000 (2011)	8,346 (2009)	27% (2009)
**Namibia**	4,251 (2010)	4,475 (2010)	8% (2007)
**Niger**	7,000 (2010)	8,840 (2006)	76% (2006)
**Nigeria**	49,000 (2011)	46,698 (2011)	78% (2011)
**Rwanda**	50,000 (2012)	43,400 (2010)	27% (2008)
**Sao Tome and Principe**	305 (2010)	300 (2002)	29% (2010)
**Senegal**	7,550 (2009)	7,090 (2008)	39% (2008)
**Seychelles**	432 (2010)	400 (2009)	27% (2010, of male prisoners only)
**Sierra Leone**	2,237 (2010)	1,975 (2009)	49% (2009)
**Somalia**	N/A	N/A	N/A
**South Africa**	157,375 (2011)	118,154 (2011)	30% (2011)
**Swaziland**	2,628 (2009)	2,838 (2009)	28% (2009)
**Togo**	4,116 (2010)	N/A	80% (2010)
**Uganda**	31,683 (2011)	14,334 (2011)	54% (2011)
**United Republic of Tanzania**	38,353^b^ (2011)	27,653 (2009)	51% (2011)
**Zambia**	16,666 (2010)	7,500 (2009)	35% (2005)
**Zimbabwe**	15,000 (2010)	17,000 (2010)	30% (2010)

a Among the 22 (of 33) prisons under government control.

b Not including Zanzibar.

N/A, not available.

One reason for overcrowding is extended pretrial detention. Half or more of the prison population consists of remanded prisoners (who have not been convicted) in Angola, Benin, Burundi, Cameroon, Chad, Comoros, Congo, Liberia, Mali, Niger, Nigeria, Togo, Uganda, and Tanzania (Table 1). Lengthy pretrial detention can also violate international human rights obligations, including prohibitions on mixing remanded prisoners with convicted prisoners [10], and have serious health consequences [11].

This policy proposal discusses how criminal justice system failures and limited financial resources present barriers to reducing HIV and TB transmission in prisons and how “structural rights” interventions focused upon criminal justice system reform are needed to guarantee detainees' human rights and health. To better understand structural barriers to HIV and TB prevention in African prisons, we conducted a survey of prison commissioners and medical directors in East and Southern African countries with high HIV and TB rates. Written surveys were sent via email, fax, or post to prison authorities in the 18 states that are members of the Southern African Development Community (SADC) and East African Community (EAC). Ten surveys were returned (from Burundi, Kenya, Malawi, Mauritius, South Africa, Swaziland, Uganda, United Republic of Tanzania, Zambia, and Zimbabwe). Topics covered in the survey included size of the prison population; available health services; HIV and tuberculosis prevalence; mortality rates; donor and government funding for health services; and challenges in health care administration. Respondents were informed of the purpose of the survey and that information provided would be publicly reported. Consistent with the US Code of Federal Regulations, which exempts ethics review for surveys of elected or appointed public officials, independent ethics approval was not sought [12]. Our policy recommendations are also informed by in-depth interviews with 246 prisoners and 30 prison officers at six prisons in Zambia and 164 prisoners and 30 prison officers at 16 prisons in Uganda, reported previously [13]–[15], and a review of relevant literature.

## Criminal Justice System Failures

International law requires that individuals who are detained be brought “promptly” before a judge, and be charged or released [10]. International law further states that individuals are entitled to trial within a “reasonable” amount of time and should not, as a general rule, be detained while awaiting trial [10].

Under Ugandan law, a suspect must be charged by a court within 48 hours of arrest [16]. Yet, 85% of prisoners interviewed in Uganda had not been brought before a court for an initial appearance within 48 hours [14]. Despite a Zambian law that inmates be brought before a judge or magistrate within 24 hours of arrest [17], only 3% of prisoners interviewed reported having seen one within that period, and one inmate reported having been detained for over 3 years before he first saw a judge [13].

Pretrial detention is routine, and even after an initial appearance, remandees are often held for years pending trial. In Uganda, the average wait is estimated at 1 year and 3 months for capital offences, such as defilement, murder, aggravated robbery, and rape [18], and researchers interviewed a remanded prisoner who had been imprisoned for 9 years awaiting resolution of his trial [14]. One prisoner in Zambia, now convicted, reported being held 10 years pretrial [13].

Compounding these injustices is the fact that, in some cases, detentions are entirely arbitrary. In Zambia, for example, the police and the Drug Enforcement Commission enjoy broad powers under Zambian law and reportedly arrest and hold numerous alleged family members, friends, and by-standers as “co-conspirators” when their primary targets cannot be found [13]. In Uganda, media reports have alleged that wide-ranging police sweeps of people in slum areas of Kampala have been driven by the prison authorities' desires to have free manpower for prison farms and to contract out prison labor to private landowners [14].

Lack of non-custodial sentencing, restrictions on the use of parole, and delays in appeals further contribute to overcrowding. In Uganda, use of community service as an alternative to sentencing is increasing, but is inconsistently applied and highly dependent on the magistrate [14]. In Zambia, only inmates with longer sentences—those who have been found guilty of more serious crimes—are eligible for parole [13]. In both countries, prisoners wait for years for a decision on their appeals [13],[14].

## Limited Resources for Health in African Prisons

Nearly all African prison administrators in our survey cited inadequate funding as the most significant challenge to their ability to deliver health care. Prison authorities in Tanzania also cited insufficient numbers of qualified medical personnel; Swazi authorities cited poor infrastructure for health, as well as absence of a medical officer trained in HIV treatment; Zimbabwean authorities cited inadequate equipment, physical infrastructure, and training of health personnel in TB and HIV management; South African authorities cited shortages of health care professionals and lack of appropriate facilities for the management of communicable diseases; Malawian officials cited a shortage of medical equipment and drugs; and Mauritian prison authorities noted insufficient health staff. Furthermore, a recently published analysis found that, between 2003 and 2010, only one of the ten countries in the Southern African region and four of the ten countries in the East African region with Global Fund-supported TB programs included TB interventions within prison settings [19].

International human rights law requires that states maintain adequate prison conditions and provide a minimum level of health care to individuals in detention [20]; care must also be at least equivalent to that available to the general population [10],[21]–[23]. Despite these protections, our research found significant gaps in the availability of HIV- and TB-related prevention and care in Zambia and Uganda [13],[14]. For example, although prisons expanded HIV testing in both countries in recent years, access to testing is limited and treatment is inadequate. In 2010, the Zambia Prisons Service employed only 14 health staff—including one physician—to serve its 16,666 prisoners across 86 prisons, and had no prison-based antiretroviral therapy (ART) or TB treatment facilities [13]. Of Uganda's 223 prisons, only one prison hospital provided prison-based ART and TB treatment [14].

Treatment in community-based facilities is theoretically possible at some prisons; however, access to care is frequently controlled by medically untrained prison guards. Lack of adequate prison staff, transportation, and fuel for the transfer of sick prisoners, as well as security fears, keep inmates from accessing medical care outside of prisons, in some cases for weeks after they fall ill [13]–[15]. In Uganda, we found that prisoners requiring transfer to facilities with HIV or TB treatment may be denied or delayed care, and instead forced into a brutal hard labor system. Prisoners receiving ART or TB treatment were sometimes transferred away from the one prison-based facility where they could receive medical care in order to ease congestion or provide labor on farms [14].

The promise of expanded ART “treatment as prevention” (TasP) approaches to impact community-level HIV transmission [6] has been documented in British Columbia, Canada [24], San Francisco [25], and Taiwan [26], and has the potential to reduce TB risk at both an individual and population level [7]. TasP is the focus of a large number of ongoing research trials [27], and has also won the attention of programmatic and political leaders. However, the promise of TasP, as well as pledges to provide universal access to HIV prevention, treatment, care, and support [28], cannot be realized if prisoners are unable to initiate, or sustain, treatment while detained, and to be effectively referred to treatment programs upon release. Reducing the number of individuals in pretrial detention or detained for minor offenses can greatly reduce the burden on prison systems to expand health care and ensure continuity of treatment for incoming and released detainees.

## Addressing Structural Barriers to HIV and TB Control through Criminal Justice Reforms

“Structural-rights” interventions [29] can complement traditional public health interventions and address criminal justice failures that contribute to overcrowding, and in turn to HIV and TB transmission. Reducing arbitrary and extended pretrial detention, for example, is a cost-effective criminal justice measure [30], and large-scale prisoner releases, as recently announced in Zambia [31], can immediately address overcrowding. Interventions such as reforming bail guidelines, restricting overly broad police authority to detain “co-conspirators,” expanding the availability of community service and parole programs, increasing the numbers of judges, and improving access to legal representation, can reduce prison populations in a sustained manner. These interventions would likely be cost-effective as well, and prevent both injustice and prison-based HIV and TB infection by reducing the number of people unnecessarily jailed or waiting years for trial. Greater funding is also needed for HIV and TB prevention, but in the absence of criminal justice system reform, funding alone may be ineffective (Box 1).

**Box 1. Scaling up HIV Prevention in Prisons without Addressing Structural Barriers**

Under Global Fund Round 8, Zambia was granted 3.7 billion kwacha (almost US$800,000) over 2 years for HIV prevention in prisons [34]. HIV prevalence in Zambian prisons was last measured at 27% and spreads, in part, through sexual relationships [35]. For a Prisons Service with a health budget of 0 kwacha (US$0) in 2009, and 200 million kwacha (US$42,210) in 2010, such an infusion of funding could be game-changing [13]. But how is US$800,000 to be spent effectively on HIV prevention when prison authorities ban condoms, citing the criminalization of same-sex sexual conduct?

One approach could be to expand voluntary HIV counselling and testing. Some research has shown that people who know their HIV-positive status are more likely to change behavior [36],[37], and international guidance on HIV in prisons recommends voluntary counseling and testing [38]. But the effectiveness of counseling and testing as HIV prevention is limited while prisoners are subject to rape and coercive sex (when sex is traded by the most vulnerable prisoners for food or soap), and where the criminalization of same-sex relationships, and a related ban on condoms, constrain risk reduction in consensual sex. The Prisons Service reported spending most of the Global Fund grant on trainings, bicycles, and a new computer.

Criminal justice system reform is not a panacea, of course. Factors other than overcrowding and pretrial detention also require attention. Inadequate nutrition is a cause of ill health among prisoners [32] and can contribute to poor adherence to ART and TB therapy and to TB infection and disease [33]. Rape and coercive sexual relationships must be addressed by taking steps to end sexual violence in prison and ensuring the provision of basic necessities to prisoners in accordance with international standards. Not all prison systems that are overcrowded have high rates of remand prisoners (Table 1), and improving prison facilities, many unchanged since colonial periods, is also essential.

## Conclusion

While nearly all prisoners return to the community, many serve multiple short sentences, cycling in and out of prison. Visitors and prison officers also link prisons to the community, bridging prison health and public health. Both increased resources for health, as well as structural interventions addressing criminal justice failures, are necessary to address HIV and TB and advance prisoner and public health. African governments have a responsibility to address the life-threatening conditions in prisons, which are contrary to international law and standards, and to improve prisoners' access to justice. Both African governments and international health donors should fund justice initiatives and other structural interventions to address HIV and TB in prisons and the general population in Africa.
